# Deficiency of HMGN2 enhances antibacterial activity of macrophages by promoting H3 histone modification-mediated CD14/iNOS expression

**DOI:** 10.3389/fimmu.2025.1621440

**Published:** 2025-10-30

**Authors:** Zhen Yang, Xiao Zhang, Chaoqun Liu, Ning Huang, Yan Teng, Junming Miao

**Affiliations:** ^1^ Department of Ophthalmology, Affiliated Hospital of North Sichuan Medical College, Nanchong, China; ^2^ Department of Optometry, North Sichuan Medical College, Nanchong, China; ^3^ Department of Neurology, Sichuan Provincial People’s Hospital, School of Medicine, University of Electronic Science and Technology of China, Chengdu, China; ^4^ Department of Pathophysiology, West China College of Basic and Forensic Medicine, Sichuan University, Chengdu, China

**Keywords:** HMGN2, macrophage, CD14, epigenetic modification, nitric oxide

## Abstract

High-mobility group nucleosomal-binding domain 2 (HMGN2) is a widely recognized chromatin-structural protein within the nucleus of eukaryotes. It has been demonstrated to be implicated in immune responses during bacterial infection. Nevertheless, the regulatory mechanism of HMGN2 in the antibacterial process of macrophages remains unclear. In this research, distinct alterations in HMGN2 expression in macrophages were observed subsequent to microbial stimulation. To investigate the role of HMGN2 in macrophages during infection, the CRISPR-Cas9 technology was employed to construct an HMGN2-knockout RAW264.7 cell line. It was verified that HMGN2 knockout could significantly enhance the bactericidal and phagocytic capabilities of macrophages. The mechanistic investigation revealed that cluster of differentiation 14 (CD14) was transcriptionally promoted in HMGN2-knockout macrophages. HMGN2 knockout regulates CD14 expression by augmenting histone epigenetic modification levels on the CD14 gene promoter, including H3K4me3, H3K9ac, and H3K27ac. Moreover, HMGN2 knockout can activate the CD14-mediated mitogen-activated protein kinase (MAPK) signaling pathways to facilitate nitric oxide (NO) production. This study uncovers a crucial role of HMGN2 in the macrophage-mediated host immune response. HMGN2 is anticipated to serve as a therapeutic target for the treatment of infectious diseases.

## Graphical Abstract

Diagram Comparing the Effects of HMGN2-Wild Type (HMGN2-WT) and HMGN2-Deficiency on Macrophage Bactericidal Function. The left panel shows that under bacterial infection, CD14 mediates the activation of the MAPK pathway in macrophages, which further induces the phosphorylation of p38, JNK, and ERK. Ultimately, this regulates the occurrence of phagocytosis and the production of nitric oxide (NO), both of which are involved in the bactericidal process. The right panel shows the cellular changes in the state of HMGN2-deficiency: under the same infection conditions, histone modification in the CD14 gene promoter region of macrophages is altered, promoting the upregulation of CD14 expression. Enhanced CD14 expression drives the improvement of cellular phagocytic function and mediates the increased expression level of NO by activating the MAPK signaling pathway, ultimately enhancing the bactericidal capacity of macrophages. Green arrows indicate increased activity or upregulated expression level.

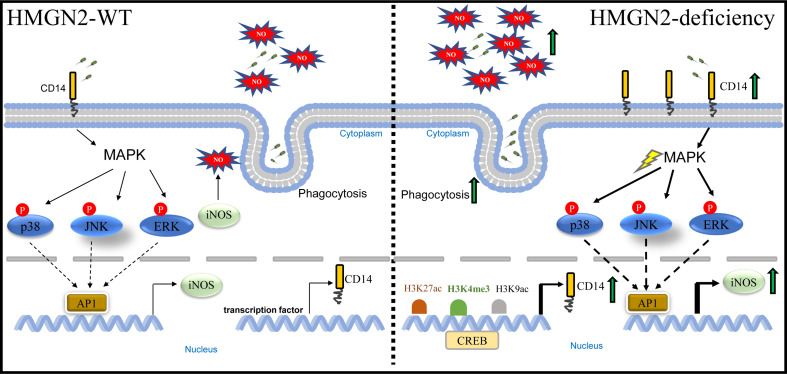

## Highlights

- The deletion of HMGN2 significantly enhances the phagocytic and bacterial killing capabilities of macrophages.
- The deletion of HMGN2 facilitates histone modifications at the promoter of the CD14 gene. This includes elevating the levels of H3K4me3, H3K9ac, and H3K27ac, thereby enhancing the transcription of CD14.
- The deletion of HMGN2 activates the CD14-mediated MAPK signaling pathway, thereby enhancing the production of NO.

## Introduction

High mobility group nucleosomal binding domain 2 (HMGN2) belongs to the HMGN family. This family consists of a group of non-histone proteins that are widely present in the nuclei of eukaryotic cells. The molecular structure of HMGN2 consists of three parts: the nuclear- localization signal domain, the nucleosome-binding domain, and the chromatin-regulating domain. Guided by the nuclear-localization signal domain, HMGN2 can bind to the nucleosome via the nucleosome-binding domain. Subsequently, the chromatin-regulating domain will exert its function to affect gene transcriptional activity (1–3).

HMGN2 is considered to have a dual function in regulating gene expression. The regulation can be either promotional or inhibitory, depending on diverse pathophysiological conditions (1–3). HMGN2 is indispensable for innate immune function. It has been reported that HMGN2 influences the adhesion and invasion of pathogenic microorganisms in lung or bladder epithelial cells by regulating factors such as microRNAs, the membrane receptor integrins, the Mitogen-activated protein kinase (MAPK) signal pathway, the Nuclear factor kappa-light-chain-enhancer of activated B cells (NF-κB) signaling pathway, oxidative stress, cell autophagy, and cytoskeletal-related proteins. Thus, it safeguards epithelial cells from infections by *Escherichia coli* and other gram-negative bacteria (4–14). HMGN2 also exerts a role in macrophages. Specifically, the knockdown of HMGN2 by small RNA can impact macrophage polarization and impede the cellular survival of *Mycobacterium tuberculosis* (15). HMGN2 can modulate pyocyanin-induced autophagy by influencing its enrichment at the unc-51 like autophagy activating kinase 1 (*Ulk1)* gene promoter via acetylation modification (16). Nevertheless, the function of HMGN2 in macrophages during infection and the associated regulatory mechanism remain ambiguous.

Although the signaling pathways activated by the stimulation of pathogenic microorganisms in macrophages have been comprehensively elaborated and explained, it remains incompletely understood how macrophages maintain persistent gene expression and undergo reprogramming in the presence of a persistent stimulus (17, 18). Epigenetic modification plays a crucial role in regulating the immune defense of macrophages. According to recent research, over 12,000 enhancers can be reprogrammed when mouse macrophages are transplanted into a new microenvironment. During the reprogramming of macrophages, histone epigenetic modification, DNA epigenetic modification, mRNA nuclear retention, and translation all undergo extensive changes (19, 20). It has been demonstrated that HMGN2 influences gene transcription by modulating chromosome architecture and histone modification, including methylation and acetylation, to regulate the interaction between transcription factors and chromatin (1, 3, 21). Therefore, we hypothesize that HMGN2 plays a role in the process of macrophage reprogramming by affecting the function of transcriptional regulatory elements at the chromatin level during infection.

In the current study, we verified that HMGN2 is indispensable for the immune response of macrophages during bacterial infection. A reduction in HMGN2 expression in macrophages during the infection process can enhance phagocytic activity and bacterial clearance by facilitating Cluster of differentiation 14 (CD14)-mediated nitric oxide (NO) release. Our findings indicate that HMGN2 holds significant potential as a therapeutic target for clinical infectious diseases.

## Materials and methods

### Materials

All materials, kits, and antibodies utilized in this research are listed in **Table 1**.

**Table 1:** The information of materials used in this study.

Materials	Source	Identifier
Antibodies		
Rabbit anti-CD14	Proteintech	Cat#17000-1-AP, RRID:AB_2074048
Rabbit anti-HMGN2	Cell Signaling Technology	Cat# 9437, RRID:AB_10949505
Rabbit anti-H3K27me3	ABclonal	Cat# A2363, RRID:AB_2756439
Rabbit anti-H3K27ac	ABclonal	Cat# A7253, RRID:AB_2767797
Rabbit anti-H3K4me3	Cell Signaling Technology	Cat# 9751, RRID:AB_2616028
Rabbit anti-H3K9ac	ABclonal	Cat# A7255, RRID:AB_2737400
Rabbit anti-H3	Cell Signaling Technology	Cat# 4620, RRID:AB_1904005
Rabbit anti-IgG	ABclonal	Cat# AC005, RRID:AB_2771930
Rabbit anti-CREB	Cell Signaling Technology	Cat# 9197, RRID:AB_331277
Rabbit anti-β-actin	ABclonal	Cat# AC028, RRID:AB_2769861
Rabbit anti-iNOS	Cell Signaling Technology	Cat# 13120, RRID:AB_2687529
APC-Cy7-antiCD11b	Biolegend	Cat# 101225, RRID:AB_830641
PE-anti-F4/80	Cell Signaling Technology	Cat# 88154, RRID:AB_2800116
Chemicals, Peptides, and Recombinant Proteins		
Lipopolysaccharides	Millipore Sigma	Cat #L2880
Puromycin Dihydrochloride	Beyotime	Cat #ST551
L-NAME hydrochloride	MedChemExpress	Cat #HY-18729A
SB203580	MedChemExpress	Cat #HY-10256
SP600125	MedChemExpress	Cat #HY-12041
PD98059	MedChemExpress	Cat #HY-12028
T-5224	MedChemExpress	Cat #HY-12270
Critical Commercial kits		
EndoFree Maxi Plasmid Kit	TIANGEN	Cat #DP117
Gel Extraction Kit	Qiagen	Cat #28706
Total RNA Isolation Kit	Sangon Biotech	Cat #B511321
SYBR qPCR Master Mix	Vazyme	Cat #Q711
cDNA Reverse Transcription Kit	Vazyme	Cat #R122
One Step Cloning Kit	Vazyme	Cat #C115
Chemiluminescence detection kit	Millipore Sigma	Cat #WBULS0100
Total Nitric Oxide Assay Kit	Beyotime	Cat #S0023
CD14 ELISA kit	Rndsystems	Cat #MC140
Cell Counting Kit-8	Apexbio	Cat # K1018
Mammalian Membrane Protein Extraction Kit	Transgen	Cat # DE301
Double-Luciferase Reporter Assay Kit	Transgen	Cat # FR201
SimpleChIP® Enzymatic Chromatin IP Kit	Cell Signaling Technology	Cat # 9003
pHrodo™ Green E. coli BioParticles™ Conjugate	Thermofisher scientific	Cat # P35366
Experimental Models: Cell Lines, Animals, Bacterial Strains		
Mouse RAW264.7 cell line	Sichuan University Huang Lab	N/A
Escherichia coli ATCC 19138	Sichuan University Huang Lab	ATCC 19138
Escherichia coli J96	Sichuan University Huang Lab	J96
Klebsiella pneumonia K33	Sichuan University Huang Lab	K33
Software and Algorithms		
Graphpad prism 7.0	GraphPad Software	https://www.graphpad.com
Image Lab	Biorad	https://www.bio-rad.com

### Mice

C57BL/6 HMGN2 knockout (HMGN2-/-) mice were generated by Beijing Biocytogen Co., Ltd. This knockout model eliminates HMGN2 expression at the genome-wide level. C57BL/6 Wildtype (WT) mice were obtained from Chengdu Dossy Experimental Animals Co., Ltd. All mice were maintained under standard conditions at the animal center of University of Electronic Science and Technology of China. All necessary measures were taken to avoid or minimize discomfort, pain, or distress to the animals. Animal procedures were approved by the Institutional Animal Care and Use Committee of University of Electronic Science and Technology of China (NO.1061420210305013).

### Cell line and bacteria strains

Mus musculus macrophage RAW264.7 cells were purchased from ATCC and stored in our lab, cells were cultured with DMEM medium containing 10% fetal bovine serum, 100 μg/ml streptomycin and 100 U/ml penicillin at 37°C in a standard cell incubator with 5% CO_2_. Bacteria strains *Escherichia coli* 19138, *Klebsiella Pneumoniae* K33, *Uropathogenic Escherichia coli* J96 are preserved in our lab. Bacteria were cultured in Luria-Bertani (LB) broth for overnight to reach the logarithmic phase. The concentration of bacteria suspensions was measured by the absorbance at 600nm.

### HMGN2-knockout and HMGN2/CD14 double knockout cell lines construction

Genome-editing tool clustered regularly interspaced short palindromic repeats (CRISPR)-Cas9 system was used to construct the HMGN2 gene deletion cell line. Briefly, Lenti-Crispr-V2-gRNA-hmgn2 plasmid were transfected into RAW264.7 cells with transfection reagent (poly plus) according to the product manual. The small guide RNA (sgRNA) sequence (5’-AGG CTG GCT TAC CTT CGC AGG GG-3’) was designed by https://portals.broadinstitute.org/gpp/public/analysis-tools/sgrna-design website. After 48 hours, puromycin was used to select genome-edited cells. Five days later, limiting dilution analysis was used to select the HMGN2 knockout monoclonal cell. Western blot analysis and DNA sequencing were used to identify HMGN2 knockout cells. For HMGN2/CD14 double knockout cell line construction, HMGN2 knockout cells were used following the protocol above, the sgRNA sequence 5’-CTT TCC TCG TCT AGC TCG CA-3’ was used for mouse CD14 deletion.

### Cell viability detection

Wildtype and HMGN2 knockout RAW264.7 cells were seeded into 96-well plates for 5000 cells per well and cultured in standard condition (DMEM medium, 37°C, 5% CO_2_) without any treatment for 24 hours, or treated with LPS (1μg/mL) for 24 hours. 10μL cell counting kit (CCK)-8 solution was added to each well and incubated at 37°C for two hours. The absorbance value at 450nm was measured by using a microplate analyzer and the corresponding cell number was calculated.

### Real time quantitative PCR

Total RNA was extracted using the Total RNA Isolation Kit according to the protocol provided by manufacturer. 0.5μg RNA were used for cDNA synthesis, cDNA was then applied for real time quantitative PCR amplification following the manufacturer’s instructions. Primers used in this study were listed in **Table 2**.

**Table 2:** Primers used in the study.

Gene name	Oligonucleotides	Source
β-actin Forward	GGCTGTATTCCCCTCCATCG	Tsingke Biological Technology
β-actin Reverse	CCAGTTGGTAACAATGCCATGT	Tsingke Biological Technology
iNOS Forward	GTTCTCAGCCCAACAATACAAGA	Tsingke Biological Technology
iNOS Reverse	GTGGACGGGTCGATGTCAC	Tsingke Biological Technology
CD14 Forward	CTCTGTCCTTAAAGCGGCTTAC	Tsingke Biological Technology
CD14 Reverse	GTTGCGGAGGTTCAAGATGTT	Tsingke Biological Technology
HMGN2 Forward	TGAAGGGGATGCTAAAGGAGA	Tsingke Biological Technology
HMGN2 Reverse	GTGCCTGGTCTGTTTTGGC	Tsingke Biological Technology
-5 ChIP Forward	CCGACCATGGTGAGTCAGAC	Tsingke Biological Technology
-5 ChIP Reverse	CTGCCAGACCCTGAGATTCC	Tsingke Biological Technology
-50 ChIP Forward	ACTGAAGCCTTTCTCGGAGC	Tsingke Biological Technology
-50 ChIP Reverse	CCACCCCAAGACTGTCTGAC	Tsingke Biological Technology
-100ChIP Forward	GCTGCCAAATTGGTCGAACA	Tsingke Biological Technology
-100ChIP Reverse	GCTCCGAGAAAGGCTTCAGT	Tsingke Biological Technology
-200 ChIP Forward	GGAAGAGAAGTGGAGACGCA	Tsingke Biological Technology
-200 ChIP Reverse	TGTAATCCTGGGGTGTCACC	Tsingke Biological Technology
-450 ChIP Forward	ACGGTGGGAAATTGCTAGCA	Tsingke Biological Technology
-450 ChIP Reverse	TCTGTTGCTCAGTCCCATTGA	Tsingke Biological Technology
-750 ChIP Forward	GAAAAAGCTGGGCATGGTGG	Tsingke Biological Technology
-750 ChIP Reverse	ATTTTCCTCCTGCCTCTGCC	Tsingke Biological Technology
-900 ChIP Forward	GGAACTCTTTTTGCAGGCCAG	Tsingke Biological Technology
-900 ChIP Reverse	TAAGAGACAGAGGCAGGCCA	Tsingke Biological Technology
-1000ChIP Forward	GATGACGATGACGACGACGA	Tsingke Biological Technology
-1000 ChIP Reverse	GCCGTCAGCATTGTCTTCATC	Tsingke Biological Technology
CREB ChIP Forward	AGGCTAATAGGAAATGACT	Tsingke Biological Technology
CREB ChIP Reverse	AGGCTAATAGGAAATGACT	Tsingke Biological Technology

### Western blot

Cells were stimulated with LPS for 24 h, and cells were washed with clean PBS for three times, then cells were lysed with RIPA buffer containing Protease inhibitors and phosphatase inhibitors for 30min at 4°C. Cell debris were removed by centrifuge. The concentration of protein was measured with BCA reagent according to the protocol. The obtained protein was then separated by SDS-PAGE and then transferred to the PVDF membrane and then incubated with specific antibodies overnight, followed by incubate with the peroxidase-conjugated goat anti rabbit secondary antibody. The immunoreactive bands were then analyzed.

### Immunofluorescence

RAW264.7 cells seeded into the slides. After being stimulated with LPS for the indicated time respectively, cells were washed three times with precooled PBS for 3 times, then fixed with 4% paraformaldehyde for 15 minutes, followed by permeation with Triton for 20 minutes, then sealed with goat serum for 30 minutes, and incubated overnight with HMGN2 (or F4/80) primary antibody. At the second day, cells were washed and then stained with fluorescent secondary antibodies for one hour. Then the nuclei were stained with DAPI solution and observed under a fluorescence microscope.

### Flow cytometry

Cells were collected and suspended in staining buffer. After three times wash, cells were counted and the F4/80, CD11b antibodies were added at optimal concentrations and incubated on ice for 20min in dark. Then cells were washed for three times and the flow cytometric analysis were performed as instructions. For HMGN2 staining, the HMGN2 primary antibody was added and incubated with cells for 30min, then cells were washed for three times and then incubated with FITC- conjugated secondary antibody for 30min. Cells were then analyzed.

### Enzyme-linked immunosorbent assay and nitric oxide detection

Shortly, the supernatant after LPS stimulation was collected, centrifuged at 12000 rpm for 10 minutes. For serum collection, blood was collected and left to rest for two hours, then centrifuged at 3500 rpm for 10 minutes. The concentration of CD14 were measured by CD14 ELISA kit according to the manufacturer instruction. The concentration of NO was measured by Total Nitric Oxide Assay Kit.

### Phagocytosis assay

One day in advance, WT and HMGN2-/- RAW264.7 cells were inoculated into 24-well plate, and the Fluorescein-conjugated pHrodo™ Red *E. coli* BioParticles (Invitrogen) were added into each well at a ratio of 1:10 (cell:particles) and mixed well. The cells were placed in incubators at 37°C for three hours. The phagocytosis ability of the cells was detected by flow cytometry and fluorescence microscopy.

### Bacterial killing assay

RAW264.7 cells were inoculated at 2×10^5^/well into a 24-well plate and then stimulated with LPS for 24 hours. bacteria (*Escherichia coli* 19138, *Klebsiella Pneumoniae* K33, *Uropathogenic Escherichia coli* J96) were added and co-incubated with RAW264.7 cells for different time and different MOI (multiplicity of infection, number ratio of bacteria to cells). Then Triton-100 was added and cells were lysed for 30 minutes, cells and fluid were next collected, diluted and plated on the LB plates. Bacterial colonies were counted.

### Dual luciferin reporting assay

Shortly, CD14 promoter was inserted in PGL3-basic plasmid which expressed Renilla luciferase. WT and HMGN2-/- RAW264.7 cells were inoculated into 96-well plate at 2×10^4^, cells were co-transfected with PGL3-mCD14 promoter plasmid and internal reference PRL-SV40 plasmid. 48 hours later, cells were stimulated with LPS for 24h, then the fluorescence values of Renilla luciferase and firefly luciferase were detected and calculated according to the protocol.

### Chromatin immunoprecipitation

ChIP assay was applied and performed as reported previously (9). Shortly, initially, conduct formaldehyde cross-linking on WT and HMGN2 knockout cells to fix the protein-DNA complexes. Subsequently, lyse the cells and fragment the chromatin DNA into 150-900bp small pieces through sonication. Next, introduce H3K4me3, H3K27ac, H3K9ac, H3K27me3, or IgG antibodies for immunoprecipitation to capture the target protein-DNA complexes. Incorporate Protein A/G beads to bind the antibody-target protein-DNA complexes and collect them via precipitation. Wash the precipitated complexes to eliminate non-specific binding and acquire the enriched target protein-DNA complexes. Then, reverse the cross-linking and purify the enriched DNA fragments. Take 2% of the Input DNA, the DNA after immunoprecipitation (IP) with the target protein antibody (H3K4me3, H3K27ac, H3K9ac, H3K27me3), and the DNA after IP with the IgG antibody as templates respectively. Add the ChIP primers corresponding to the target genes to be detected (**Table 2**) respectively, conduct quantitative PCR reactions, and obtain their respective Ct values. Calculate the ChIP enrichment efficiency according to the following formula: Signal relative to Input = 2% × 2(Ct [2%Input Sample]– Ct [IP Sample]).

### *In vivo* bacterial killing assay

For *in vivo* infection, 2×10^5^ bacteria (*Escherichia coli* 19138) were injected intraperitoneally (*i.p.*), 24 hours later, abdominal cells are collected by collecting abdominal flushing fluid, the lung, spleen, liver were anatomically separated out and grinded into single cell suspension, and then plated into LB-plate, the relative survival of bacteria was calculated using the plate colony count method (n=5).

### Statistical analysis

All data in this study were processed by means of GraphPad Prism 7 and are presented as the mean ± standard deviation (SD). All data were analyzed through two-tailed unpaired Student’s t-tests for comparisons between two groups. For multiple comparisons, one- or two-way Analysis of Variances (ANOVA) followed by Bonferroni *post hoc* tests were employed. When P < 0.05, the difference between groups was regarded as statistically significant. For all cellular results, a minimum of three independent and repeated experiments were carried out. In the animal experiments, at least four mice were utilized.

## Results

### The expression of HMGN2 is decreased in macrophages stimulated by bacteria

**Figure 1:**
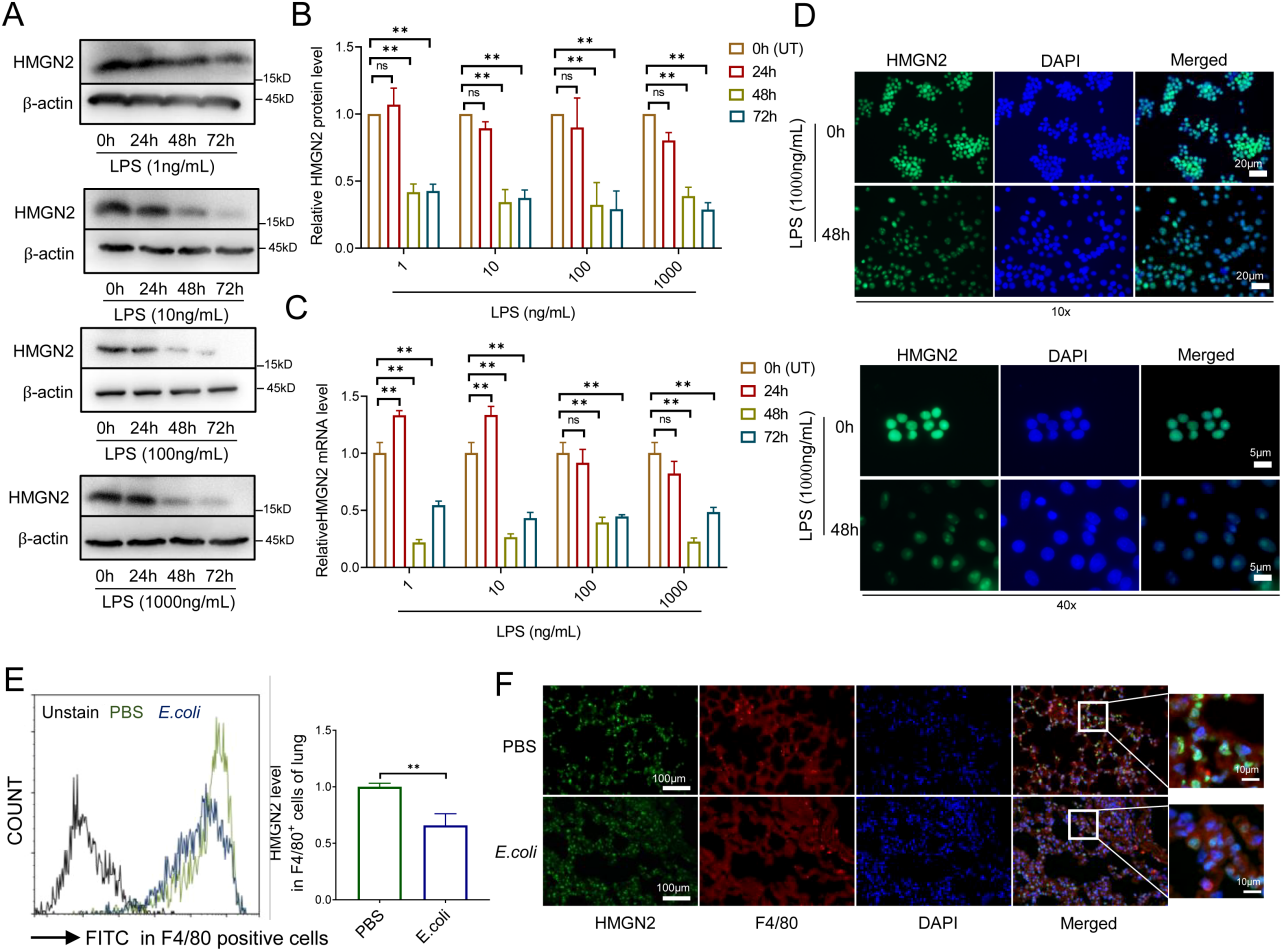
Heterogeneous expression of HMGN2 in macrophages upon stimulation with microbe. **(A–C)** RAW264.7 mouse macrophages were stimulated by different concentration of LPS for 24, 48 or 72 hours, protein expression **(A, B)** and mRNA level **(C)** of HMGN2 were measured by western blot and RT-qPCR respectively. All data are represented as mean ± SD. “**” indicates p<0.01 compared to untreated cells (0 hour, UT), three independent and repeated experiments were conducted. **(D)** RAW264.7 mouse macrophages were treated with 100ng/mL of LPS for 48 hours, HMGN2 expression was detected by immunofluorescence (HMGN2: green). The upper panel consists of the images captured under the 10× objective lens, whereas the bottom panel contains the images acquired under the 40× objective lens. **(E, F)** Wild-type mice were infected with 1×10^5^ colony-forming unit (CFU) *E*. *coli* intratracheally, lung tissue was anatomically separate out 24 hours later, HMGN2 expression in lung macrophages was detected by flowcytometry **(E)** and immunofluorescence **(F)**. Data are represented as mean ± SD of three mice, “*” indicates p<0.05.

The expression of HMGN2 varies in different immune microenvironments, it can be either promoted or inhibited to regulate various physiological or pathological reactions (1, 9, 22). To investigate the impact of bacterial infection on HMGN2 expression in macrophages, we stimulated the mouse RAW264.7 macrophage cell line with *Escherichia coli* (*E. coli*) endotoxin lipopolysaccharide (LPS). Subsequently, we detected HMGN2 expression through western blot analysis (**Figures 1A, B**), RT-qPCR (**Figure 1C**), and immunofluorescence assays (**Figure 1D**). The results indicated that the expression of HMGN2 was significantly decreased by more than half in LPS-stimulated macrophages after 48 or 72 hours. However, we also discovered that short-term LPS stimulation (1ng/ml and 10 ng/ml for 24 hours) could promote the mRNA expression of HMGN2, suggesting a complex regulatory mechanism of HMGN2 in macrophages during LPS stimulation.

We further established a murine model of lung infection to verify the alteration in HMGN2 expression within bacteria-infected macrophages *in vivo*. Specifically, we surgically administered an intratracheal injection of *E. coli* strain 19138 into the lung tissues of C57BL/6 mice. Twenty-four hours post-injection, the expression of HMGN2 in macrophages of mouse lung tissue was detected via flow cytometry (**Figure 1E**) and immunofluorescence (**Figure 1F**).

The findings revealed that the expression of HMGN2 in macrophages of *E. coli*-infected lung tissue was 35% lower compared to that in uninfected mice (treated with phosphate - buffered saline, PBS). These results suggest that HMGN2 may play a crucial role in macrophages infected by bacteria.

In subsequent experiments, the cells were treated with 1μg/mL of LPS for 24 hours. This treatment corresponds to the maximum concentration and the longest treatment duration that do not induce alterations in the expression of HMGN2. As such, it effectively circumvents any interference stemming from changes in the basal level of HMGN2.

### HMGN2 deficiency enhances the bacterial-killing activity of macrophages

**Figure 2:**
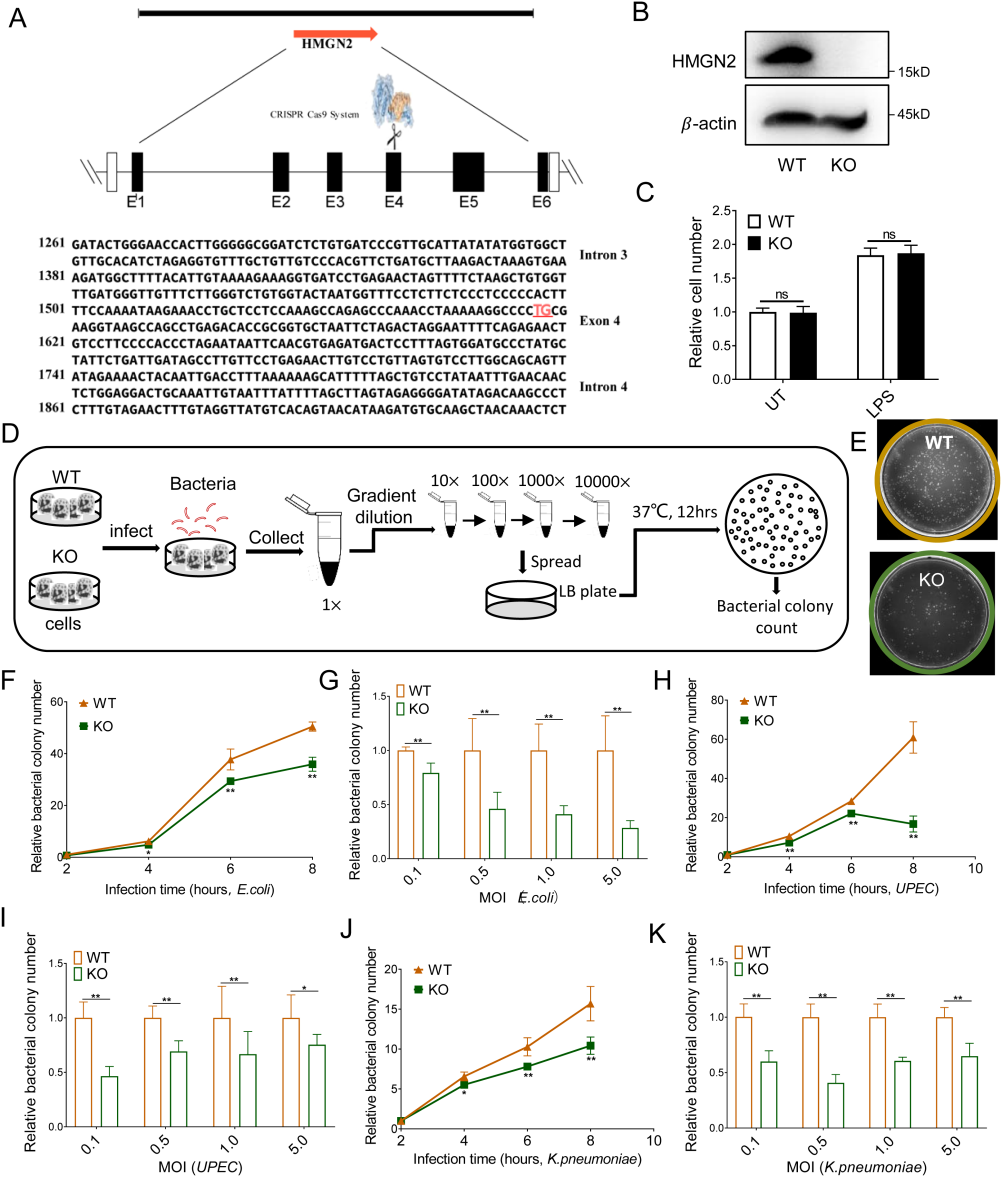
HMGN2 deficiency enhances the antibacterial capabilities of macrophages. **(A)** Schematic diagram showing that HMGN2 knockout RAW264.7 cell was constructed by CRISPR-Cas9 technology. **(B)** Western blot assay showing the expression of HMGN2 in wild-type (WT) and HMGN2 knockout (KO) RAW264.7 macrophages. **(C)** WT and HMGN2-KO macrophages were normally cultured for 24 hours (Ctrl) or treated with LPS (1μg/mL) for 24 hours, cell number was measured by CCK-8 assay. **(D)** Schematic diagram of *in vitro* killing assay. The cells were incubated with live bacteria for indicated time, the culture medium was subjected to serial dilution, spread onto LB agar plates, and then incubated overnight at 37°C. Live bacteria were quantified in terms of colony-forming units (CFU). The bacterial killing capabilities were reflected by the alterations in the number of CFUs. **(E)** It was shown the representative LB agar plates of WT and HMGN2-KO groups. **(F, G)** *In vitro* killing assay of *E*. *coli* 19138 by RAW264.7 macrophages. **(F)** RAW264.7 cells were infected by *E*. *coli* at indicated infected time and the number of CFUs were counted. MOI is multiplicity of infection which represents number ratio of bacteria to cells, MOI=5 in this experiment. **(G)** RAW264.7 cells were infected by *E*. *coli* at indicated MOI for 8 hours and the number of CFUs were counted. **(H, I)** *In vitro* killing of *UPEC* by RAW264.7 macrophages. **(J, K)** *In vitro* killing of *K*. *pneumoniae* by RAW264.7 macrophages. All data are represented as mean ± SD. “ns” indicates no statistical significance, “*” indicates p<0.05, “**” indicates p<0.01 compared to WT group, three independent and repeated experiments were conducted.

To investigate the role of HMGN2 in macrophages, we utilized clustered regularly interspaced short palindromic repeats (CRISPR)-Cas9 technology to establish a HMGN2 knockout mouse RAW264.7 cell line. In the HMGN2 knockout cells, a deletion of two bases on the fourth exon of the HMGN2 gene (indicated in red font) was induced (**Figure 2A**). The western blot results demonstrated the absence of HMGN2 in the knockout cells (**Figure 2B**). Additionally, the CCK-8 assay results indicated that there was no significant change in the cell number when compared with wild-type RAW264.7 cells (WT) after 24 hours of culture under physiological (UT) or LPS stimulation conditions (**Figure 2C**).

Macrophages were incubated with live *E. coli* strain 19138. The impact of HMGN2 knockout on the bactericidal capacity of macrophages was detected via the *in-vitro* bacterial killing assay (**Figure 2D**). Following incubation with macrophages, the number of live bacteria remaining in the HMGN2-KO group was significantly lower than that in the WT group (**Figures 2E–G**), indicating an enhanced bactericidal capacity of HMGN2-KO RAW264.7 macrophages. In addition to *E. coli*, the influence of HMGN2 knockout on the bactericidal capacity of macrophages was also investigated using other common pathogens, including *uropathogenic Escherichia coli* (*UPEC*) and *Klebsiella pneumoniae (K. pneumoniae)*. The results were consistent with those obtained for the *E. coli* strain. HMGN2-KO macrophages demonstrated stronger bactericidal capabilities during *UPEC* (**Figures 2H, I**) and *K. pneumoniae* infection (**Figures 2J, K**), with fewer live bacteria remaining. These findings suggest that HMGN2 knockout can enhance the bacterial-killing capacity of RAW264.7 macrophages.

### HMGN2 deficiency enhances CD14 expression

**Figure 3:**
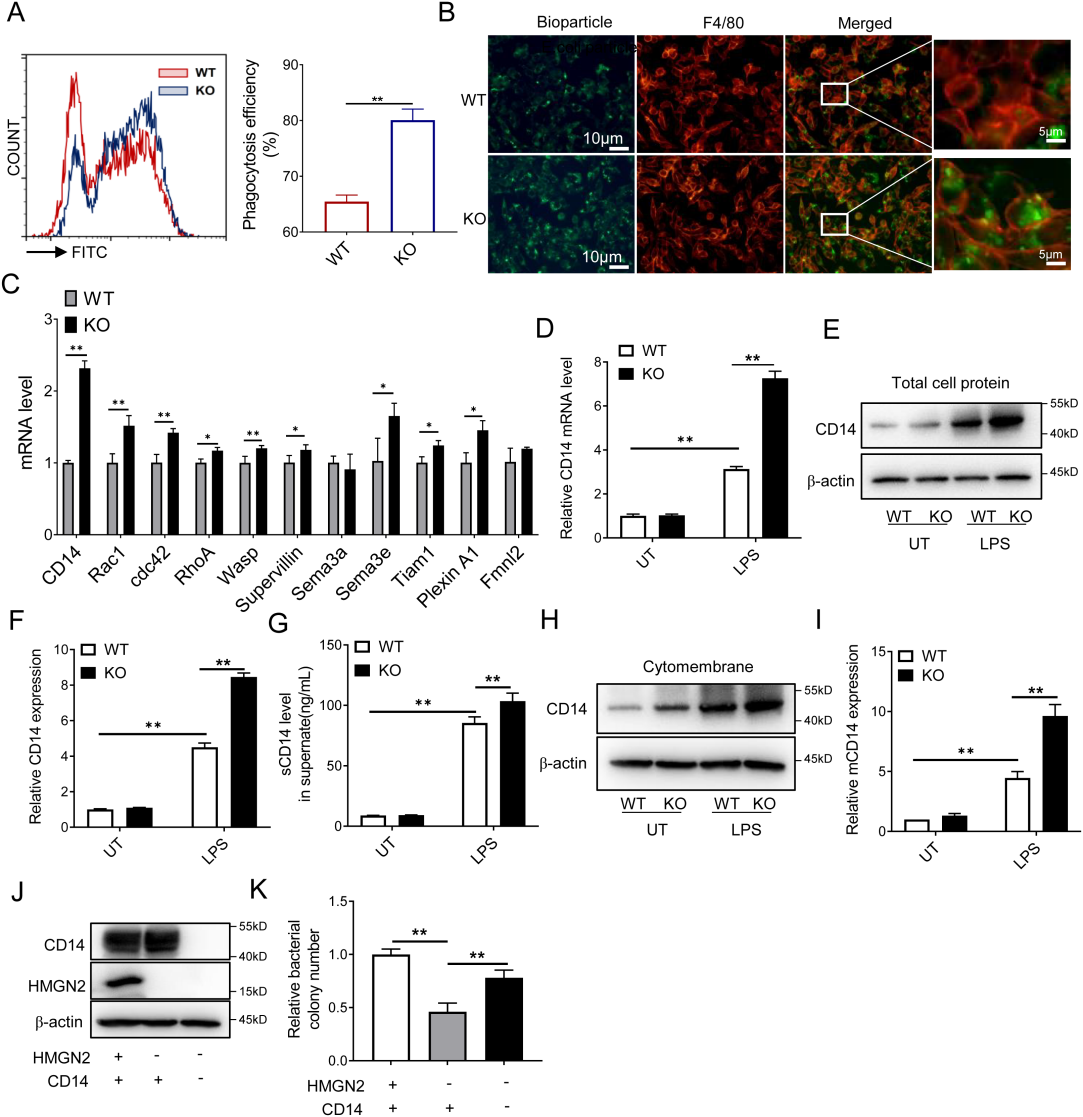
HMGN2 deficiency enhances CD14 expression. **(A)** the *In vitro* phagocytic capacity of HMGN2-KO macrophages was measured by using fluorescein conjugated Phrodo™ *E. coli* bioparticles (green) under flowcytometry. Phagocytosis efficiency was expressed as the mean fluorescence intensity. **(B)** *In vitro* phagocytic capacity of HMGN2-KO macrophages (PE-F4/80, red) was also assessed under microscope. **(C)** The expression levels of multiple genes associated with macrophage phagocytic function were measured by RT-qPCR upon LPS stimulation (1mg/mL for 24 hours). **(D-F)** WT and HMGN2-KO macrophages were normally cultured for 24 hours (Ctrl) or treated with LPS (1mg/mL) for 24 hours, CD14 expression was measured by RT-qPCR **(D)** and western blot **(E, F)**. **(G)** WT and HMGN2-KO macrophages were treated the same as **(D)**, cell culture supernatant was collected and the level of soluble CD14 (sCD14) was measured by ELISA. **(H-I)** WT and HMGN2-KO macrophages were treated the same as **(D)**, protein level of membrane CD14 (mCD14) was measured by western blot. **(J)** HMGN2 and CD14 double knockout RAW264.7 macrophages (HMGN2-/- CD14-/-) was constructed based on HMGN2 knockout cell (HMGN2-/-). Western blot showing the expression of HMGN2 and CD14 in cells. **(K)** *In vitro* killing of E. coli 19138 by HMGN2-/- and HMGN2-/-/CD14-/- RAW264.7 macrophages. RAW264.7 cells were infected by E. coli for 8 hours (MOI=5), the bacterial killing capabilities were reflected by the changes of CFUs. All data are represented as mean ± SD of more than three independent repeated experiments. “*” indicates p<0.05, “**” indicates pp<0.01 compared to indicated group.

Macrophages represent a crucial class of phagocytic cells. To explore whether HMGN2 knockout affects the bacterial-killing capacity of macrophages by influencing their phagocytic function, we detected the phagocytic ability of HMGN2-knockout cells. We found that the phagocytic ability of HMGN2-KO cells was significantly stronger than that of WT cells (**Figure 3A**) by using fluorescent *E. coli* biological particles and flow cytometry. A similar phenotype was also observed in the result of immunofluorescence result (**Figure 3B**). To identify the downstream target genes of HMGN2 that govern the phagocytic function of macrophages, we then analyzed the gene expression associated with phagocytosis and discovered that most phagocytic molecules were upregulated in HMGN2-knockout macrophages upon LPS stimulation. These molecules included *Cd14*, Rac family small GTPase 1*(Rac1)*, cell division cycle 42 *(Cdc42)*, Ras homolog family member A *(RhoA)*, WASP sctin nucleation promoting factor *(Wasp), Supervillin*, Semaphorin 3E *(Sema3e)*, TIAM Rac1 associated GEF 1 *(Tiam1), and PlexinA1*. Among them, CD14 was the gene with the most significant upregulation, showing more than a two-fold increase in HMGN2-knockout macrophages (**Figure 3C**).

CD14 is a crucial component of phagocytes (23–25). Subsequently, we further employed RT - qPCR and western blot techniques to investigate the impact of HMGN2 knockout on the expression of CD14 in unstimulated (UT) and LPS-stimulated RAW264.7 macrophages. The RT-qPCR results indicated that the mRNA level of CD14 in LPS-stimulated cells was significantly elevated by approximately 3.6 times compared with the UT group. Moreover, the CD14 expression in LPS - stimulated HMGN2-knockout cells was increased by twofold compared with LPS-stimulated WT cells (**Figure 3D**). The western blot results were consistent with those of RT - qPCR. Specifically, the protein level of CD14 in LPS - stimulated HMGN2-KO cells was increased by approximately 1.5 times compared with the LPS-stimulated WT group (**Figures 3E, F**).

As a pattern recognition receptor, CD14 exists in two distinct forms. One is soluble CD14 (sCD14), which is released from cells and dissolved in the extracellular fluid. The other is membranous CD14 (mCD14), which is located in the cell membrane. Although the working patterns of sCD14 and mCD14 differ slightly, both can regulate similar immune functions of macrophages. These functions include mediating bacterial phagocytosis and binding to LPS to activate downstream signaling pathways. Both sCD14 and mCD14 play crucial roles in the immune functions of macrophages during bacterial infection (24–26).

To investigate which type of CD14 is influenced by HMGN2, the cell culture supernatant was collected for the detection of the sCD14 level via the ELISA assay. The results indicated that the sCD14 level in the supernatant of LPS-stimulated HMGN2-KO cells were 1.2-fold higher than that in the supernatant of LPS-stimulated WT cells (**Figure 3G**). Meanwhile, we isolated the membrane protein of macrophages. It was found that the expression level of mCD14 was significantly elevated by 2.1-fold in LPS-stimulated HMGN2 knockout macrophages (**Figures 3H, I**). These results indicate that HMGN2 knockout can enhance the expression of both soluble and membrane CD14.

To verify whether CD14 is implicated in the HMGN2 - mediated bactericidal processes, we utilized CRISPR-Cas9 technology to establish the HMGN2-CD14 double-knockout RAW264.7 cell line (HMGN2^-/-^/CD14 ^-/-^) based on HMGN2-KO cells (**Figure 3J**). The impact of HMGN2^-/-^/CD14 ^-/-^ on the bactericidal function of macrophages was detected via an *in-vitro* bacterial-killing assay. As shown in **Figure 3K**, the number of live bacteria remaining in the HMGN2^-/-^/CD14 ^-/-^ cells was higher than that in the HMGN2 knockout cells (**Figure 3K**). This indicates that CD14 is involved in the regulation of the bactericidal function of RAW264.7 cells mediated by HMGN2.

### HMGN2 deficiency promotes CD14 expression through modulating the modification of H3 histone

**Figure 4:**
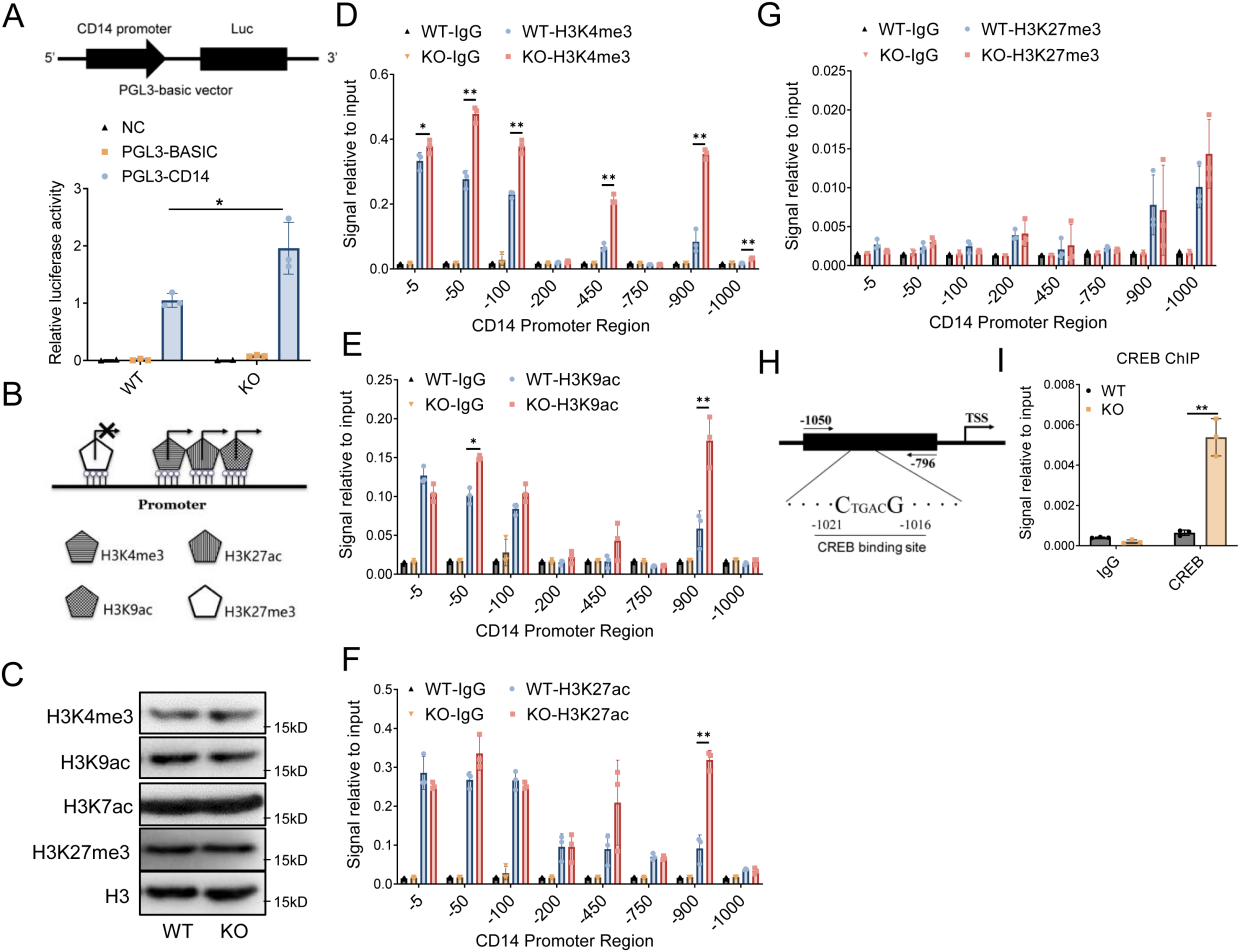
HMGN2 deficiency promotes CD14 expression through modulating the modification of H3 histone. **(A)** WT and HMGN2-KO RAW264.7 macrophages were transfected with PGL3 luciferase reporter plasmid inserted with CD14 promoter region (PGL3-CD14 promoter), luciferase activity was measured 24 hours after transfection. Empty PGL3-basic vector without CD14 promoter (PGL3-basic vector) was used as empty control, negative control indicated the macrophages without transfection. **(B)** The schematic diagram showing the common histone modifications on promoter region. **(C)** WT and HMGN2-KO macrophages were treated with LPS (1mg/mL) for 24 hours, total protein levels of H3K4me3, H3K9ac, H3K27ac and H3K27me3 were measured by western blot. **(D-G)** ChIP assay showing the recruitment of H3K4me3, H3K9ac, H3K27ac and H3K27me3 at the desired regions, the relative occupancy is the ratio of immunoprecipitated DNA to input DNA. **(H)** The schematic diagram of CREB binding motifs on promoters of CD14. The primers for ChIP assay were designed for about 6070 bp up-stream or down-stream of CREB binding motifs. **(I)** ChIP assay showing the CREB recruitment at the desired regions shown in **(H)**, the relative occupancy is the ratio of immunoprecipitated CREB to input DNA. All data are represented as mean ± SD. “*” indicates p<0.05, “**” indicates p<0.01 compared to WT group, three independent and repeated experiments were conducted.

As a chromatin structural protein, HMGN2 has been reported to regulate gene expression by influencing the histone modification of transcriptional regulatory elements, such as promoters and enhancers (1). In order to study the mechanism of HMGN2 on CD14 expression, we initially explored the regulatory effect of HMGN2 knockout on the CD14 promoter by dual luciferase reporter system (**Figure 4A**). The pGL3 plasmid inserted with the CD14 promoter sequence was transfected into WT and HMGN2-knockout macrophages. Significantly higher luciferase activity was detected in the HMGN2 knockout macrophages. Specifically, under the stimulation of LPS, the luciferase activity in HMGN2-knockout macrophages were nearly twice that of WT cells. This result demonstrates that the transcription efficiency of the CD14 promoter in RAW264.7 cells was enhanced after HMGN2 knockout.

Histone epigenetic modification represents one of the crucial mechanisms by which HMGN2 regulates the efficiency of gene transcription. This includes modifications such as H3K4me3, H3K27ac, H3K9ac, and H3K27me3. Elevated levels of H3K4me3, H3K27ac, and H3K9ac enhance transcription efficiency, while an increased level of H3K27me3 inhibits transcription efficiency (**Figure 4B**) (27). Initially, we analyzed the protein levels of these four histone modifications via Western blot. It was found that the protein levels of total H3K4me3, H3K9ac, H3K27Ac, and H3K27me3 in HMGN2 - knockout macrophages exhibited no significant alterations compared to those in WT cells (**Figure 4C**). Subsequently, chromatin immunoprecipitation (ChIP) was employed to detect the impact of HMGN2 knockout on histone modification in the CD14 promoter region. We discovered that, following LPS stimulation, the enrichment levels of H3K4me3, H3K27ac, and H3K9ac at multiple sites in HMGN2 - knockout macrophages were increased compared to those in WT cells (**Figures 4D, G**). Notably, H3K4me3 demonstrated the most significant elevation, with increased enrichment around positions -5, -50, -100, -450, -900, and -1000 upstream of the transcription start site (**Figure 4D**). H3K9ac also showed increased enrichment at positions -50 and -900 upstream of the transcription start site (**Figure 4E**), and H3K27ac exhibited increased enrichment at position -900 upstream of the transcription start site (**Figure 4F**). These findings indicate that, under the stimulation of LPS, HMGN2 knockout enhances CD14 transcriptional efficiency by promoting histone modifications, including H3K4me3, H3K27ac, and H3K9ac, in the CD14 promoter region.

The regulation of gene transcription level through histone modification is accomplished by altering the interaction between the promoter and transcription factors. It has been reported that the cAMP-response element binding protein (CREB), which serves as an important transcription regulator for promoting CD14 gene transcription, can enhance the CD14 transcription level by interacting with the CD14 promoter region (28). To further confirm whether the knockout of HMGN2 can enhance the transcription regulation efficiency of the CD14 gene by facilitating the enrichment of the transcription factor CREB in the promoter region near the -900 and -1000 sites, a ChIP assay was utilized to detect the enrichment level of CREB in the CD14 promoter region (**Figure 4H**). The results indicated that in LPS-stimulated macrophages, HMGN2-KO cells demonstrated an 83.4-fold higher enrichment level of CREB transcription factors on the CD14 promoter. Specifically, this enrichment level was five times that of the WT group (**Figure 4I**).

### HMGN2 deficiency facilitates the CD14-mediated expression of inducible nitric oxide synthase

In addition to phagocytosis, CD14, serving as a crucial co-receptor of LPS, is also capable of activating intracellular signals via downstream signaling pathways such as the MAPK and NF-κB. Consequently, it regulates the transcription of immune - related genes (24, 25, 29). Hence, the higher CD14 expression induced by HMGN2 knockout may also facilitate the high expression of antimicrobial substances, thereby enhancing the bactericidal effect of macrophages.

**Figure 5:**
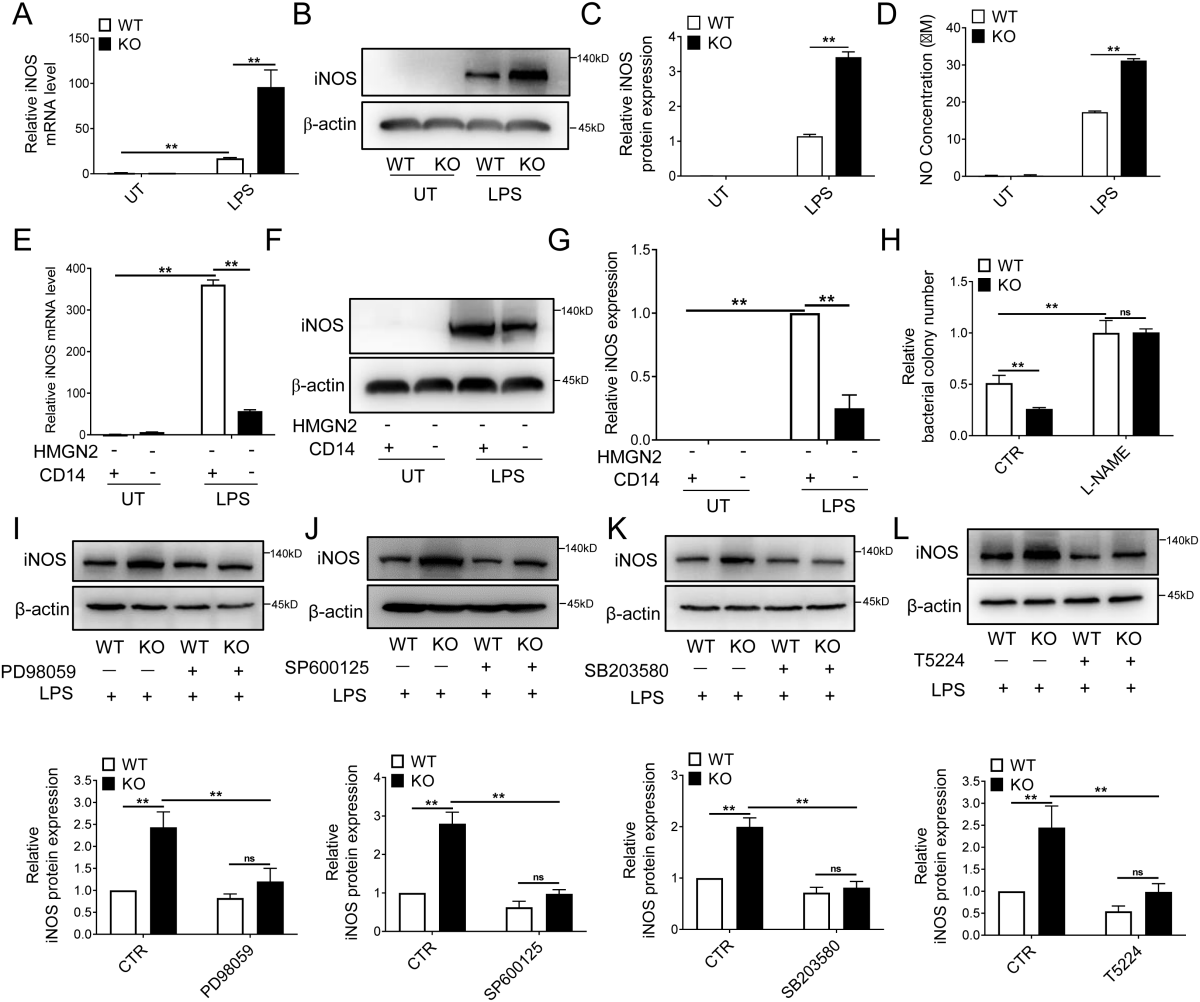
HMGN2 deficiency facilitates the CD14-mediated expression of iNOS. **(A–C)** WT and HMGN2-KO macrophages were treated with LPS (1μg/mL) for 24 hours, iNOS expression was measured by RT-qPCR **(A)** and western blot **(B, C)**. **(D)** WT and HMGN2-KO macrophages were treated the same as **(A)**, cell culture supernatant was collected, and the level of NO was measured by Total Nitric Oxide Assay Kit. **(E–G)** HMGN2^-/-^ and HMGN2^-/-^/CD14^-/-^ RAW264.7 macrophages were treated with LPS (1μg/mL) for 24 hours, iNOS expression was measured by RT-qPCR **(E)** and western blot **(F, G)**. **(H)** WT and HMGN2-KO RAW264.7 macrophage was pre-treated with iNOS inhibitor L-NAME (200μM) for three hours, solvent treated cells (CTR) was set as control. *In vitro* killing of *E*. *coli* 19138 by WT and HMGN2-KO RAW264.7 macrophages was then examined the same as **Figure 2D**. **(I–L)** WT and HMGN2-KO RAW264.7 macrophage was pre-treated with ERK inhibitor PD98059 (30μM), JNK inhibitor SP600125 (10μM), P38 inhibitor SB203580 (10μM) and AP-1 inhibitor T5224 (10μM) for two hours, followed by LPS stimulation (1μg/mL, 24 hours), the level of iNOS was detected by western blot. All data are represented as mean ± SD. “ns” indicates no statistical significance, “**” indicates p<0.01 compared to indicated group, three independent and repeated experiments were conducted.

Nitric oxide (NO) is a crucial molecule in the bactericidal function of macrophages. It can be released from activated macrophages to the extracellular environment and directly interact with pathogenic microorganisms to exert bactericidal effects (30). We found that no production was significantly enhanced in HMGN2-knockout macrophages. RT-qPCR showed that the iNOS expression in HMGN2-knockout macrophages was about 18.5 times higher than that in the WT (**Figure 5A**). Western blot yielded similar results. After 24-hour LPS stimulation, the iNOS level in HMGN2-knockout macrophages was nearly three times that in the WT (**Figures 5B, C**). Additionally, the NO level in the HMGN2-knockout macrophage culture medium was nearly 1.8 times that of the WT (**Figure 5D**). These results suggest that HMGN2 knockout enhances iNOS expression and promotes NO production.

Next, we used HMGN2/CD14 double-knockout RAW264.7 macrophages to detect whether HMGN2 promotes the expression of iNOS through CD14. The RT-qPCR results (**Figure 5E**) and western blot results (**Figures 5F, G**) both showed that, after knocking out CD14 on the basis of HMGN2 knockout, the originally elevated iNOS expression was significantly inhibited. This indicates that CD14 knockout can reverse the HMGN2 knockout-induced elevated iNOS expression. In other words, CD14 is involved in the regulation of HMGN2-mediated iNOS expression.

To verify whether NO is the effector molecule in the enhanced bactericidal ability of macrophages resulting from HMGN2 knockout, cells were treated with the iNOS inhibitor L-NAME. Subsequently, an *in-vitro* bacterial killing assay was employed to detect the impact of L-NAME on the bactericidal function of macrophages. As shown in **Figure 5H**, in the control group (CTR, without inhibitor), HMGN2 knockout enhanced the bactericidal function of macrophages. However, after treatment with L-NAME, this enhancing effect was neutralized. This indicates that NO may be a crucial effector molecule in the bactericidal ability of macrophages promoted by HMGN2 knockout.

Under inflammatory conditions, CD14-mediated iNOS expression is regulated via MAPK and NF-κB pathways (24, 25, 31). To confirm the involvement of the MAPK pathway in HMGN2-mediated NO production, macrophages were treated with extracellular-regulated protein kinases (ERK) inhibitor (PD98059), c-Jun N-terminal kinase (JNK) inhibitor (SP600125), p38 mitogen - activated protein kinase (P38) inhibitor (SB203580), and an MAPK downstream transcription factor activator protein-1 (AP-1) inhibitor (T-5224). Western blot was used to detect iNOS expression, and the results showed that inhibitor treatment reversed the increased iNOS expression induced by HMGN2 knockout (**Figures 5I–L**). We also employed inhibitors of the NF-κB signaling pathway and did not observe similar reverse outcomes (not shown). These findings indicate that the MAPK signaling pathway is crucial for HMGN2-mediated regulation of macrophage NO production.

### HMGN2 deficiency enhances the *in vivo* bacterial clearance ability of mice

**Figure 6:**
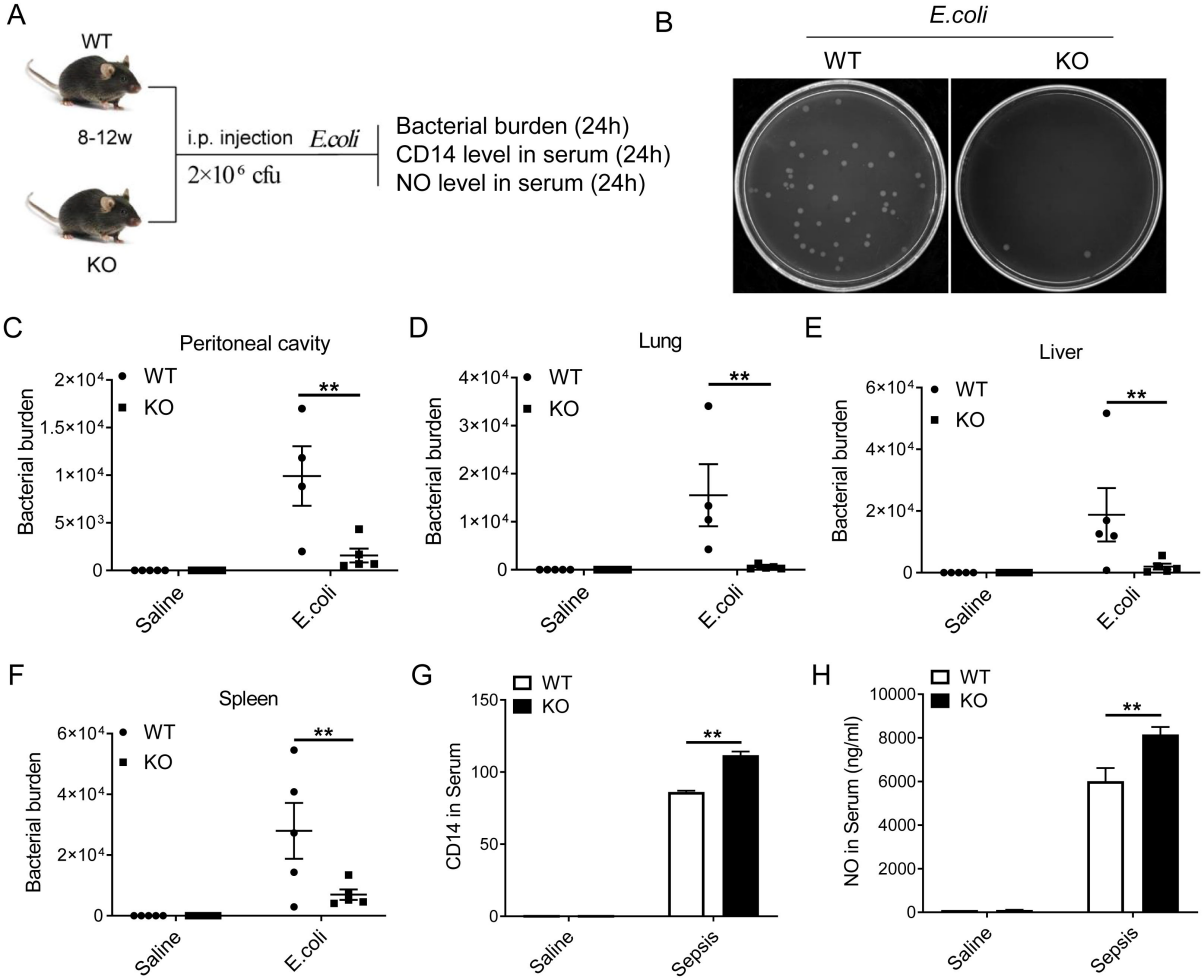
HMGN2 deficiency enhances the *in vivo* bacterial clearance ability of mice. **(A)** Experimental scheme. WT and HMGN2 knockout mice were challenged with *E. coli* 19138 (2×10^6^ CFUs) intraperitoneally (*i.p.*). **(B, C)** Bacterial burden were quantified as CFUs remaining in peritoneal cavity (n=5). **(D, F)** Total CFUs remaining in the indicated organs 24 hours after *E. coli* challenge (n=5). **(G)** CD14 level in serum was detected by using ELISA assay 24 hours after *E. coli* injection (n=4). **(H)** NO level in serum was detected by using Total Nitric Oxide Assay Kit 24 hours after *E. coli* injection (n=4). All data are represented as mean ± SD. “**” indicates p<0.01 compared to WT.

We established a murine bacterial infection model to investigate the impact of HMGN2 knockout on the ability of mice to eliminate pathogenic microorganisms. *E. coli* was intraperitoneally injected into WT and HMGN2-knockout mice. Twenty - four hours after infection, the peritoneal lavage fluid was collected, and the lung, liver, and spleen tissues of the mice were dissected. The number of live bacteria in each tissue was detected using the *in-vivo* bacterial killing assay (**Figure 6A**). The results demonstrated that, compared with WT mice, the number of live bacteria remaining in the peritoneal cavity of HMGN2 knockout mice was decreased by 85% (**Figures 6B, C**). There was scarcely any live *E. coli* remaining in the lung tissue of HMGN2 knockout mice (**Figure 6D**). The number of live bacteria remaining in the liver of HMGN2 knockout mice was decreased by 90% (**Figure 6E**), and the number of live bacteria remaining in the spleen of HMGN2 knockout mice was decreased by 80% (**Figure 6F**). These findings indicated that HMGN2 knockout significantly enhanced the clearance of infected bacteria in mice.

To verify whether the knockout of HMGN2 promotes the production of CD14 and NO *in vivo*, the serum was collected 24 hours after bacterial treatment. The serum levels of CD14 and NO were determined using the ELISA method and the Griess Reagent method, respectively. It was found that the levels of CD14 (**Figure 6G**) and NO (**Figure 6H**) in HMGN2-knockout mice were significantly higher than those in WT mice. This indicates that the knockout of HMGN2 also promotes the production of CD14 and NO *in vivo*.

## Discussion

Phagocytes not only eliminate pathogenic microorganisms through phagocytosis but also exert a bactericidal effect extracellularly by releasing a series of antimicrobial substances, such as reactive oxygen species (ROS), nitric oxide (NO), antimicrobial peptides, and other substances (32). This study reveals that during bacterial infection, the decrease in HMGN2 levels precisely regulates the acetylation and methylation modifications of histone H3, driving a significant upregulation of CD14 gene transcription. This epigenetic regulatory mechanism further activates multiple antibacterial functions of macrophages. It not only enhances the uptake efficiency of pathogens by phagocytes but also promotes the expression of iNOS, thus establishing a robust extracellular bactericidal barrier.

These findings not only fill the theoretical gap in the understanding of macrophage immune response mechanisms but also open new directions for clinical intervention in infectious diseases. For therapeutic applications, future research could develop small-molecule inhibitors, epigenetic regulatory drugs, or cellular gene-editing therapies based on HMGN2 to enhance innate immunity, which can be combined with antibiotics to tackle drug-resistant bacterial infections. Clinically, HMGN2 and its downstream molecules may serve as novel biomarkers for infection diagnosis, disease monitoring, and prognosis assessment. They could also be targets for vaccine adjuvant optimization and inflammatory storm regulation, with great significance and translational prospects. However, issues remain, like how to directionally regulate HMGN2 in macrophages.

This study has limitations as it didn’t use other knockout cell lines targeting different loci for repeated verification or conduct a rescue experiment. However, we used HMGN2 knockout mice and the *in vivo* results matched *in vitro* findings. CRISPOR tool (parameters: PAM=NGG, mismatch ≤ 2bp) prediction shows only 2 potential sgRNA off-target sites in non-coding regions. HMGN2’s role in regulating antibacterial gene expression is affirmed (1). The enhanced bactericidal ability of HMGN2 knockout cells aligns with its regulation of genes like CD14/iNOS. Although off-target effects can’t be fully excluded, existing data from gene editing verification, functional mechanism correlation, and probability analysis suggest HMGN2 is the direct phenotype regulator.

Increased intracellular HMGN2 expression was detected in lung epithelial cells infected with *E coli* and *K pneumoniae* for 2, 4, and 6 hours (9). HMGN2 expression in macrophages was also elevated at 3, 6, and 12 hours post-infection with *Mycobacterium tuberculosis* (15). In this study, HMGN2 expression in LPS-stimulated mouse RAW264.7 macrophages changed dynamically, it increased at 24 hours, then declined after 48 and 72 hours. *In vivo*, HMGN2 expression in mouse lung macrophages significantly reduced 24 hours after bacterial infection. These studies verified HMGN2 expression in immune cells is heterogeneous, depending on pathogens, tissues, and infection duration, reflecting its complexity and precision in immune defense regulation.

Based on our findings and global research progress, HMGN2 may play distinct regulatory roles in early and late LPS stimulation. This supplements the theoretical mechanism of macrophage polarization and bactericidal function. During infection, macrophages reprogram bactericidal mechanisms according to the complex immune microenvironment. At the initial LPS stimulation, macrophages secrete inflammatory factors, stimulating immune cells via autocrine/paracrine to amplify the immune response. Early increased HMGN2 expression in response to LPS may be a rapid macrophage response to external stimuli. Increased HMGN2 may boost inflammatory factor production to combat microbial invasion, and high HMGN2 can be released as an antimicrobial peptide (33–36). In this study’s focus (after 24 hours, late LPS stimulation or bacterial infection), HMGN2 expression decreased and its immune response differed. HMGN2 knockout had stronger bacteria-eliminating capacity than WT, showing HMGN2 still regulates macrophage antibacterial efficiency at the relatively late stage of infection. Also, we found that reduced HMGN2 led to decreased production of inflammatory cytokines, which may prevent excessive tissue inflammation and damage.

Concerning potential mechanisms for dynamically regulating HMGN2 during the period of infection, our review of literature reveals that HMGN2 expression has been primarily found to be regulated by microRNAs. Among these, miR-23a is currently the only microRNA demonstrated to mediate targeted inhibition of HMGN2 (37–39). Notably, studies have shown that after stimulating rats with high-concentration lipopolysaccharide (LPS, 10 mg/kg), the serum miR-23a level was significantly elevated in the LPS-treated rats (38). This inversely correlated with the changes in HMGN2 expression, indirectly suggesting a potential association. Additionally, miR-23a has been confirmed to participate in the regulatory network of macrophage immune functions, as it can suppress inflammatory responses in macrophages, thereby alleviating tissue damage and inflammatory pain (40–45).

HMGN2, a chromatin structural protein, can specifically interact with nucleosomes. Recent research indicates that it is a crucial molecule in gene transcriptional regulatory elements such as promoters and enhancers. While binding to nucleosomes, it may regulate histone H3 epigenetic modification. Over 70% of HMGN2 co-localizes with DNase I hypersensitive sites, crucial for promoter and enhancer functions (1–3, 12, 21, 46–53). The deletion of HMGN2 modifies the chromatin distribution and the remodeling of DNase I hypersensitive sites, particularly in enhancer regions, thereby influencing the expression of stress-related genes in mouse embryonic fibroblasts (54). In B cells, HMGN2 co-localizes with DNase I hypersensitive sites, its deletion reduces site density and H3K4me1/H3K27ac enrichment (55). In oligodendrocytes and pluripotent stem cells, the knockdown of HMGN2 exerts an impact on the histone epigenetic modification of the gene promoter, including H3K27me3, H3K4me3, H3K9ac, and H3K27ac (56). These studies suggest a close link between HMGN proteins and the chromatin structure in the transcriptional regulatory region.

In this research, we identified common histone modifications within gene promoter regions, such as H3K4me3, H3K9ac, H3K27ac (which promote transcription) and H3K27me3 (which inhibits transcription). In HMGN2-knockout macrophages, H3K27me3 enrichment in CD14 promoter was unchanged, but H3K4me3, H3K9ac, and H3K27ac enrichments increased significantly, especially at -900bp upstream of CD14 transcription initiation site. The -900 site may be key for HMGN2 to regulate CD14 transcription. The binding site of the transcription factor CREB is located near -900 of the CD14 promoter. This indicates that histone modification-mediated CREB enrichment might be of critical importance for HMGN2 to regulate CD14 expression.

Current research on the bactericidal mechanism of HMGN2 primarily focuses on cytoplasmic signaling pathway molecules, including microRNAs, membrane receptor integrins, oxidative stress-related proteins, autophagy-related proteins, and cytoskeletal proteins. Notably, the majority of this work has been conducted in epithelial cells (4–15). In contrast, our study investigates the molecular mechanism by which HMGN2 regulates macrophages at the chromatin level. A study reported that in macrophages, HMGN2 regulates pyocyanin-induced autophagy by affecting its enrichment at the *Ulk1* gene promoter through altering its own acetylation modification (16). However, given that HMGN2 is a nuclear protein intimately associated with chromatin structure regulation, how HMGN2 alters chromatin architecture and influences immune gene expression remains unclear. This study demonstrates that in infected macrophages for the first time, HMGN2 may reprogram immune-related gene expression by modifying H3 histone marks, enabling macrophages to adapt to specific immune microenvironments.

Our study demonstrates that HMGN2 knockout regulates the expression of multiple phagocytosis-related genes, including *Cd14*, *Rac1, Cdc42, RhoA, Wasp, Sema3e, Tiam1*, and *PlexinA1*. We conclude that CD14 likely serves as a downstream mechanism through which HMGN2 modulates macrophage phagocytosis and bactericidal functions. This conclusion is supported not only by the significant upregulation of CD14 expression following HMGN2 knockout but also by the close functional association of subsequent MAPK signaling pathway activation and NO production. This is in accordance with the biological logic chain.

mCD14 serves as a surface receptor of phagocytes. It facilitates the recognition and internalization of bacteria by macrophages, triggers downstream signaling pathways, and promotes the release of bactericidal substances. It functions as the core executor in the process of bacteria clearance by macrophages. The knockout of mCD14 will attenuate the bactericidal function and heighten the susceptibility to infection. sCD14 is a protein present in body fluids. Although it cannot independently transmit signals, it can form a complex with LPS. This complex enhances the efficiency of bacteria recognition by phagocytes, aids cells lacking mCD14 in recognizing pathogens, and induces the release of inflammatory factors. mCD14 and sCD14 establish a dual-line defense mechanism of “cell surface recognition-humoral synergistic opsonization”, mCD14 plays a dominant role in direct bactericidal activity, while sCD14 broadens the scope of the immune response. Together, they optimize the efficiency of bacteria clearance (24). This study has discovered that the expressions of both mCD14 and sCD14 were upregulated in HMGN2-knockout cells. It is hypothesized that the knockout of HMGN2 promotes the complementary and amplifying effects of the two CD14s in the process of bacteria clearance. However, additional experiments need to be devised to establish the correlation between the two CD14s and the mechanisms mediated by each of them. For instance, mutant plasmids targeting different CD14 sites can be utilized.

NO, a crucial bactericidal effector from macrophages, eliminates pathogens via free radical toxicity. iNOS is the key enzyme for NO production. Its induction is influenced by multiple pathways, with MAPK and NF-κB most studied (57). CD14, interacting with LPS, activates MAPK and NF-κB, promoting iNOS expression and NO production (24, 25). This study found HMGN2 knockout significantly promoted iNOS expression and NO production in LPS-stimulated macrophages. CD14 is involved in HMGN2’s regulation of iNOS transcription. Further study of MAPK pathways showed MAPK inhibitors reversed the effect of HMGN2 knockout on iNOS. In addition, the cells were also treated with inhibitors of the NF-κB signaling pathway (not showed in this study). The results indicated that it did not exhibit a significant reversing effect on the iNOS expression induced by HMGN2 knockout. Findings suggest MAPK may dominate HMGN2’s regulation of LPS-induced iNOS expression in macrophages.

## Data Availability

The raw data supporting the conclusions of this article will be made available by the authors, without undue reservation.
